# A multidisciplinary approach to digital mapping of dinosaurian tracksites in the Lower Cretaceous (Valanginian–Barremian) Broome Sandstone of the Dampier Peninsula, Western Australia

**DOI:** 10.7717/peerj.3013

**Published:** 2017-03-21

**Authors:** Anthony Romilio, Jorg M. Hacker, Robert Zlot, George Poropat, Michael Bosse, Steven W. Salisbury

**Affiliations:** 1 School of Biological Sciences, The University of Queensland, Brisbane, Queensland, Australia; 2 Airborne Research Australia, Flinders University of South Australia, Adelaide, South Australia, Australia; 3 Autonomous Systems, CSIRO, Brisbane, Queensland, Australia; 4 Mine Environment Imaging, CSIRO, Brisbane, Queensland, Australia; 5 Autonomous Systems Lab, ETH Zurich, Zürich, Switzerland

**Keywords:** Photogrammetry, Lidar, Lower Cretaceous, Broome Sandstone, Dinosaurian tracksites, 3D visualization

## Abstract

The abundant dinosaurian tracksites of the Lower Cretaceous (Valanginian–Barremian) Broome Sandstone of the Dampier Peninsula, Western Australia, form an important part of the West Kimberley National Heritage Place. Previous attempts to document these tracksites using traditional mapping techniques (e.g., surface overlays, transects and gridlines combined with conventional photography) have been hindered by the non-trivial challenges associated with working in this area, including, but not limited to: (1) the remoteness of many of the tracksites; (2) the occurrence of the majority of the tracksites in the intertidal zone; (3) the size and complexity of many of the tracksites, with some extending over several square kilometres. Using the historically significant and well-known dinosaurian tracksites at Minyirr (Gantheaume Point), we show how these issues can be overcome through the use of an integrated array of remote sensing tools. A combination of high-resolution aerial photography with both manned and unmanned aircraft, airborne and handheld high-resolution lidar imaging and handheld photography enabled the collection of large amounts of digital data from which 3D models of the tracksites at varying resolutions were constructed. The acquired data encompasses a very broad scale, from the sub-millimetre level that details individual tracks, to the multiple-kilometre level, which encompasses discontinuous tracksite exposures and large swathes of coastline. The former are useful for detailed ichnological work, while the latter are being employed to better understand the stratigraphic and temporal relationship between tracksites in a broader geological and palaeoecological context. These approaches and the data they can generate now provide a means through which digital conservation and temporal monitoring of the Dampier Peninsula’s dinosaurian tracksites can occur. As plans for the on-going management of the tracks in this area progress, analysis of the 3D data and 3D visualization will also likely provide an important means through which the broader public can experience these spectacular National Heritage listed landscapes.

## Introduction

Fossil tracks provide direct insight into the diversity, abundance, distribution and behaviour of extinct trace-makers. Tracks formed by dinosaurs may be preserved in isolation or in association with others. A concentration of tracks, either in the form of sequential tracks made by a single trackmaker (a trackway), multiple isolated tracks, or a combination of tracks and trackways is referred to as a tracksite. The traditional technique of documenting dinosaurian tracks and tracksites has been largely restricted to acquiring data in two dimensions. While useful for determining measurements for pace and stride, the approach provides only limited morphological information for detailed comparative work (Xing et al., 2015: Fig. 6). Interpretations of the same tracksite by different investigators may contrast depending whether 2D or 3D data is utilized (Thulborn, 1997; Romilio & Salisbury, 2014). Routine mapping techniques that involve the manual placement of markers, or covering the track surface with large transparent plastic sheets and tracing track outlines (Leonardi & Santos, 2004) are time-consuming even for small tracksites. Given that some large tracksites are known to extend over hundreds of square metres (Wings & Mallison, 2015) or even square kilometres (Lockley, 1992), such traditional methods of documentation can become impractical. Although large-scale data acquisition has been attempted at some dinosaurian tracksites in Catalonia, Spain (Bates et al., 2008), and the Rocky Mountains, USA (Breithaupt, Matthews & Noble, 2004), very large tracksites can suffer from poor documentation of individual tracks (Thulborn, Hamley & Foulkes, 1994). Often this is despite such sites having enormous potential for ichnotaxonomic investigations or insights into ichnofaunal composition and trackmaker behaviour and palaeoecology.

The representation of dinosaurian tracks as outlines is inherently fraught with subjectivity (Thulborn, 1990; Bates et al., 2008; Romilio & Salisbury, 2014). While standardized protocols are currently lacking, remote sensing technologies offer enormous potential for the rapid documentation of spatially extensive tracksites. Surface-based techniques also provide varying degrees of resolution depending on the way in which the data are acquired.

Laser scanners such as lidar (originally a portmanteau of ‘light’ and ‘radar; Ring, 1963; more comonly used as an acronym of ‘Light Detection and Ranging’; NOAA, 2015) function through the active emission of light to probe a target (Sutton, 2014). Methods include triangulation (Remondino et al., 2010), time-of-flight (Bates et al., 2008), and phase-based laser scanning (Lukeneder, Lukeneder & Gusenbaur, 2014). We are unaware of the use of the latter technique in dinosaurian track research and it will not be discussed further. Triangulation-based laser scanning has been employed in the 3D visualization of individual dinosaurian tracks, and is suitable for acquiring surface data over scales ranging from sub-millimetre to several metres (Sutton, 2014). However, interference from ambient light sources severely limits practical applications in the field (Remondino et al., 2010). Time-of-flight laser scanners are considered more appropriate for field investigations (Brodu & Lague, 2012), providing surface resolution at a sub-centimetre scale with spatial coverage of hundreds of metres (Sutton, 2014). Time-of-flight scanners may be attached to mobile devices, such as handheld instruments (Bosse & Zlot, 2013; Zlot & Bosse, 2014; Zlot et al., 2014), unmanned- (Kaul, Zlot & Bosse, 2016) and manned-aircraft (Vrbancich, Lieff & Hacker, 2011; Fieber et al., 2013), or are positioned at fixed stations that then require re-positioning to avoid problems associated with a single line-of-sight (Bates et al., 2008).

Photogrammetry differs from laser scanning in that it relies on light information that is captured passively by a camera (Sutton, 2014) and can be defined as “the art, science and technology of obtaining reliable information about physical objects and the environment through processes of recording measuring and interpreting photographic images and patterns of electromagnetic radiated energy and other phenomena” (Slama et al., 1980). In one form, the target is photographed from different perspectives, which provides enough image overlap to permit the creation of the 3D Digital Surface Model (DSM) of the target structure. Digital images are now necessary for this process although analog images (subsequently converted to a digital format) have been used for ‘historical’ photogrammetry (Falkingham, Bates & Farlow, 2014; Lallensack et al., 2015). An advantage of photogrammetry is that it is not limited to size. The technique can be broadly categorized in terms of the object-to-camera distances: close- (<300 m; ‘close’ also incorporates distances of tens of centimetres with surface precision of hundreds of micrometres) or far-range (>300 m; DeWitt & Wolf, 2000). As an alternative to, or used in conjunction with distance categories, classifications may be made with regard to the relative camera position as being either ground- or aerial-based (Mozas-Calvache et al., 2012). Close-range photogrammetry is most suitable for most palaeontological studies and can be ground-based (Breithaupt, Matthews & Noble, 2004), low-altitude manned- (Hacker, 2015) and unmanned-aircraft (see Breithaupt, Matthews & Noble, 2004; Mozas-Calvache et al., 2012).

**Figure 1:**
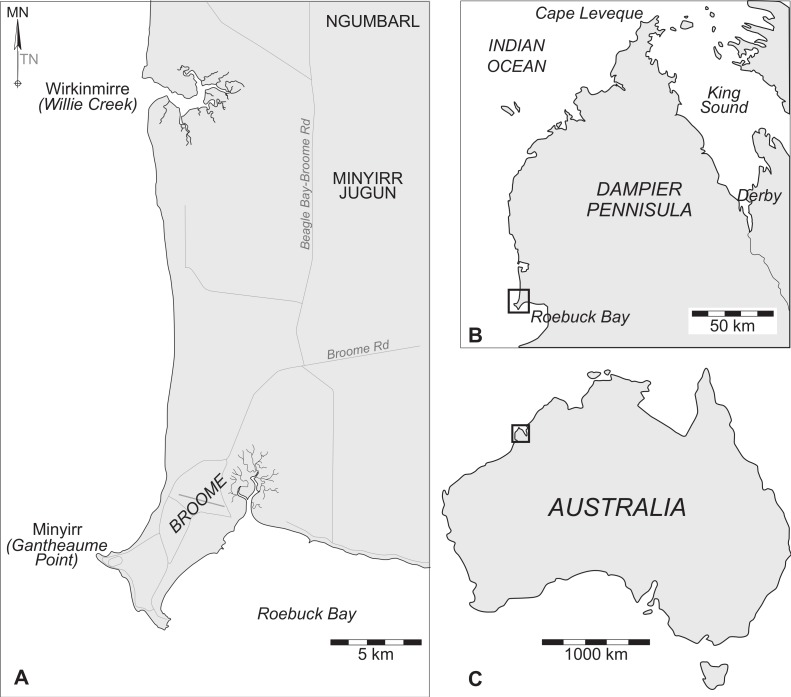
Maps showing the location of Minyirr (Gantheaume Point), south of Broome on the Dampier Peninsula in the West Kimberley, Western Australia. (A) The study area of Minyir in context with the Greater Broome area, and in relation to (B) the Dampier Peninsula and (C) Australia.

**Figure 2:**
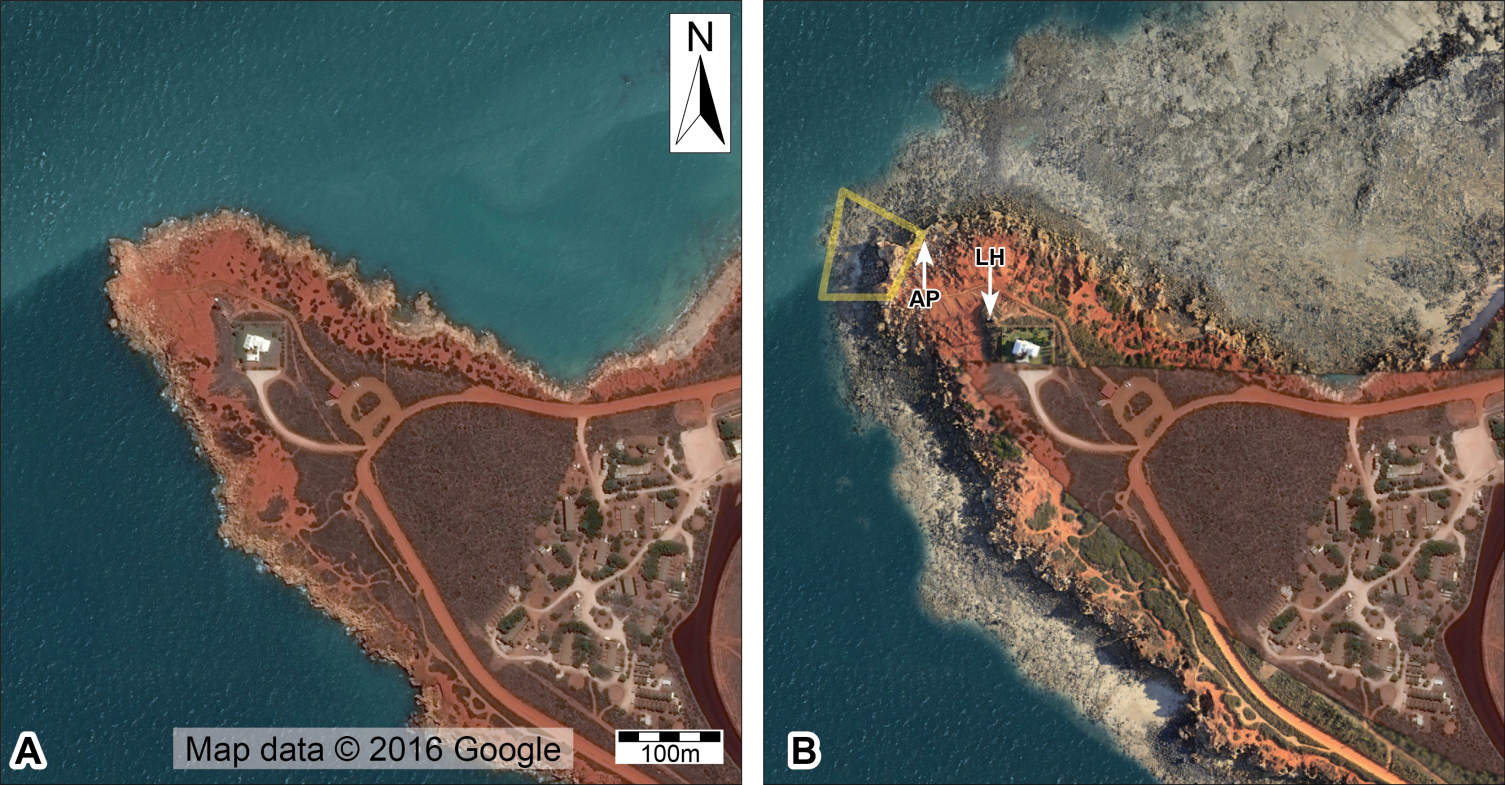
Aerial view of Minyirr (Gantheaume Point), south of Broome on the Dampier Peninsula in the West Kimberley, Western Australia. (A) High tide at ∼10 m (map image © Google) (B) Low tide at ∼0.56 m (aerial orthophotographic mosaic overlaid on map image ©  Google). Abbreviations: AP, Anastasia’s pool; LH, Lighthouse. The yellow outline indicates the portion of Minyirr (UQL-DP56) that was the focus of the current study (2.4 ha).

The Dampier Peninsula of Western Australia contains extensive coastal exposures of the Lower Cretaceous (Valanginian–Barremian) Broome Sandstone (Fig. 1), with a rich diversity of dinosaurian tracks at tracksites scattered over more than 80 km (Thulborn, Hamley & Foulkes, 1994; Long, 1998; Thulborn, 2012; Salisbury et al., 2014; Salisbury et al., in press). In addition to the scientific importance of the dinosaurian tracks, their recent inclusion in the West Kimberley National Heritage Area (Australian National Heritage List, Place ID 106063) necessitates the need for comprehensive documentation. To date, the only dinosaurian tracksite in the Broome Sandstone for which there is a published map is Minyirr (Gantheaume Point; Fig. 2). Scientific accounts of dinosaurian tracks at Minyirr date back to Glauert (1952), who never visited the site first-hand, but instead relied on Broome residents to provide illustrations and a cast of one of the tracks. Later, Colbert & Merrilees (1967) published a schematic map of a platform containing numerous tridactyl dinosaurian tracks that they described as *Megalosauropus broomensis*. While such visualizations obtained via traditional methods are useful in determining the general track morphology and the spatial arrangement of individual specimens, the resulting outlines were oversimplified representations that lack depth information and exclude much of the contextual information associated with surrounding geographical and topographical features (including other track-bearing surfaces).

Conducting research along the Dampier Peninsula is fraught with challenges, many of which may be why only one dinosaurian ichnotaxon has been described from the area (i.e., *M. broomensis*; Colbert & Merrilees, 1967). In addition to the remoteness and vastness of the tracksites, most are difficult to access primarily due to their occurrence within the intertidal zone, and being submerged by non-trivial water depths (up to 10 m) until subaerial exposure during very low tides (Fig. 2B). For some tracksites, subaerial exposure may only last several hours for a few days each year. The daily tides and periodic extreme weather conditions (e.g., heavy seas associated with summer cyclones and storms) can also greatly affect sand distribution along this stretch of coastline, periodically exposing some track-bearing rock platforms but burying others. In addition to the challenges of documentation, these issues make conservation of the National Heritage Listed tracksites very challenging.

To address these issues, and to digitally conserve the Broome Sandstone dinosaurian tracksites with high fidelity 3D digital models, we have utilized ground- and aerial-based laser scanning and photogrammetric remote sensing technologies. These permit the construction of several high-resolution digital 3D surface models that vary in scales of magnitude, from individual tracks to large swathes of coastline. Thus far, we have acquired data for in excess of 70 tracksites across 100 km of coastline. The purpose of this paper is to demonstrate how these remote sensing techniques have been used, with a focus on tracks at Minyirr. The dinosaurian tracks at Minyirr are well known locally, and the area experiences high levels of tourist visitation during the dry season (April–September) when tides permit. Minyirr is the type location of the theropod ichnotaxon *M. broomensis* (Colbert & Merrilees, 1967), and the cliff line adjacent the main track-bearing platform is also the type section of the Broome Sandstone (McWhae et al., 1956). Other ichnites at Minyirr include tracks and trackways of sauropods and ornithopods (McCrea et al., 2012; although we have not been able to confirm the latter) and flora (e.g., *Roebuckia*, *Hausmannia*, *Ptilophyllum* frond fragments and *Bucklandia* stem fragments; McLoughlin, 1996). This study represents the first attempt to comprehensively survey the dinosaurian tracks at Minyirr as currently preserved.

## Material and Methods

### Site description

Minyirr forms the northwest promontory of the Broome Peninsula, with the site’s lighthouse situated approximately 6.5 km geodesic distance southwest of Broome airport (Fig. 1). It is a named site in a song cycle that extends along the west Kimberley coast from Bunginygun (Swan Point, Cape Leveque) to Wabana (Cape Bossut, near La Grange), and then inland to the southeast over a total distance of approximately 450 km (Heritage Council of Western Australia, 1999; Major & Sarjeant, 2001). Minyirr is of high cultural significance to local indigenous groups (Bradshaw & Fry, 1989) and is a registered Aboriginal Heritage Site (K02327). In July 1801, while sailing north in the Géographie en route to the Bonaparte Archipelago, and just within sight of the coastline, French explorer Nicolas Baudin mistook the promontory and surrounding low cliffs for an island, recording it in his charts as l’île Gantheaume (Gantheaume Island) in honour of vice admiral and peer of France Honoré Joseph Antoine Gantheaume (Freycinet, 1812; Freycinet, 1815; Marchant, 1982). Following a more thorough investigation of the west Kimberley coastline by Englishman Phillip Parker King in August 1821, Baudin’s mistake was corrected and the name was changed to Gantheaume Point (King, 1827). Following the establishment of European settlement in Broome in the latter part of the 19th Century and the expansion of the region’s pearling industry and port, a lighthouse and stone cottage was constructed close to the edge of the cliff in 1905–6. The lighthouse has been rebuilt twice; first in 1917 and then again in 1984 when the existing stainless steel tower was constructed. In 1922 the keeper’s quarters were sold to Patrick Percy and his wife Anastasia. Percy had the bottom of an intertidal rock pool below the lighthouse concreted for his wife who suffered from arthritis (Burton, 2000). Both the lighthouse and Anastasia pool are now well-known local landmarks (Fig. 2), frequently visited by tourists. Unfortunately, heavy seas during January 2014 resulted in major damage to Anastasia’s pool, with large parts of the seaward wall and concrete base being broken.

**Figure 3:**
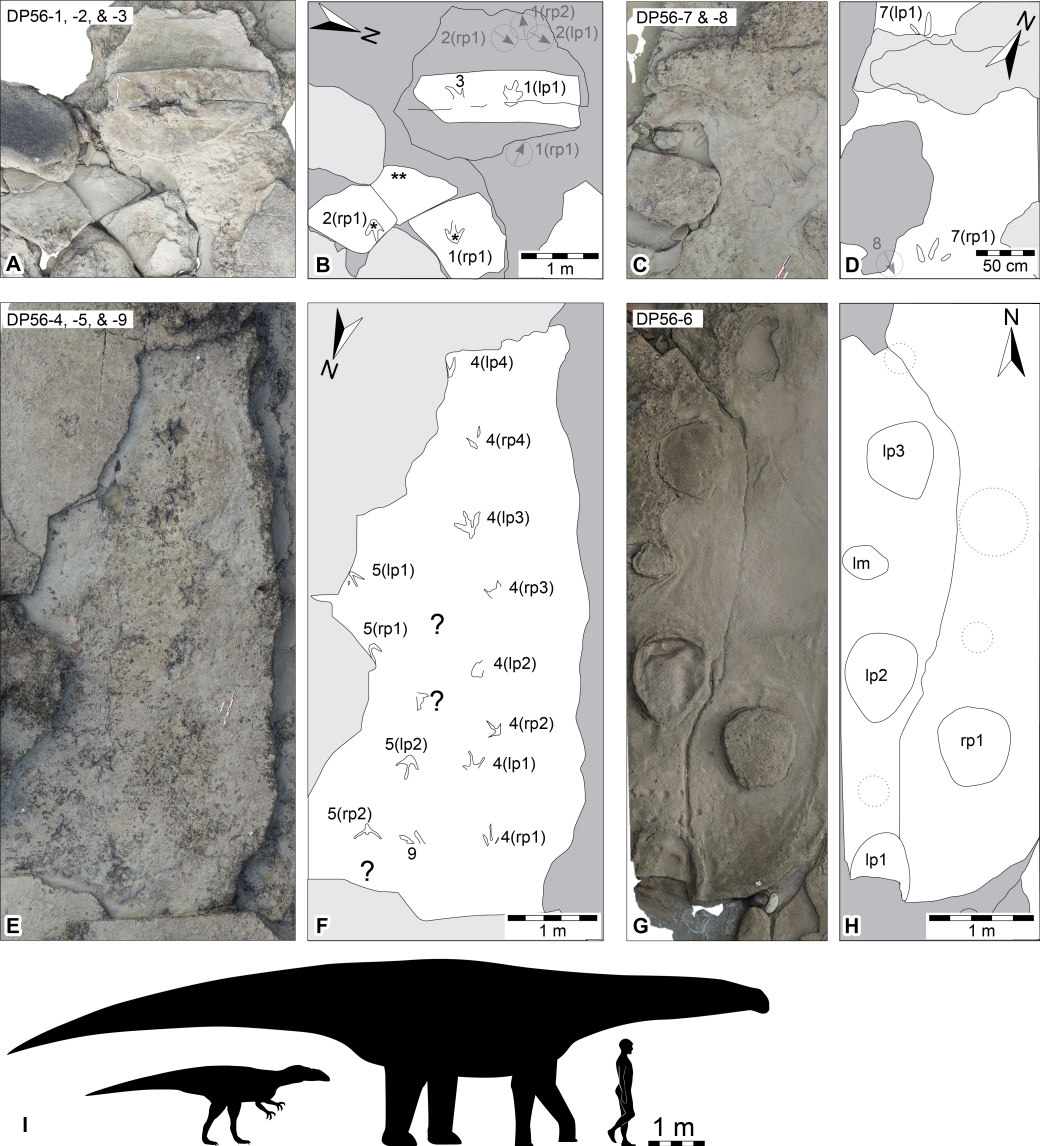
Dinosaurian track-bearing platforms in the Lower Cretaceous Broome Sandstone at Minyirr (UQL-DP56). Orthophotographic mosaics derived from ground-based photogrammetry with accompanying schematic interpretations. (A, B) *in situ* and *ex situ* theropod tracks (UQL-DP56-1, -2 and -3), orthophotographic mosaic and schematic, respectively (asterisk indicates *ex situ* track; double asterisk indicates underside of *ex situ* track surface). (C, D) *in situ* theropod tracks (UQL-DP56-7) and position of eroded track (UQL-DP56-8), orthophotographic mosaic and schematic, respectively. (E, F) *in situ* theropod trackways (UQL-DP56-4, -5 and -9), orthophotographic mosaic and schematic, respectively. (G, H) *in situ* sauropod trackway (UQL-DP56-6, orthophotographic mosaic and schematic, respectively, showing expected track positions for lost tracks (i.e., dotted circles). (I) silhouettes of hypothetical theropod and sauropod to indicate the relative trackmaker size compared with that of 1.8 m human. Grey arrow within dash-stroked circle indicates orientation and relative *in situ* track position prior to 2015. Within trackway numbering ‘l’ and ‘r’ refer to left and right impressions, respectively. Arrow indicates magnetic north (true north is approximately 2°E).

**Figure 4:**
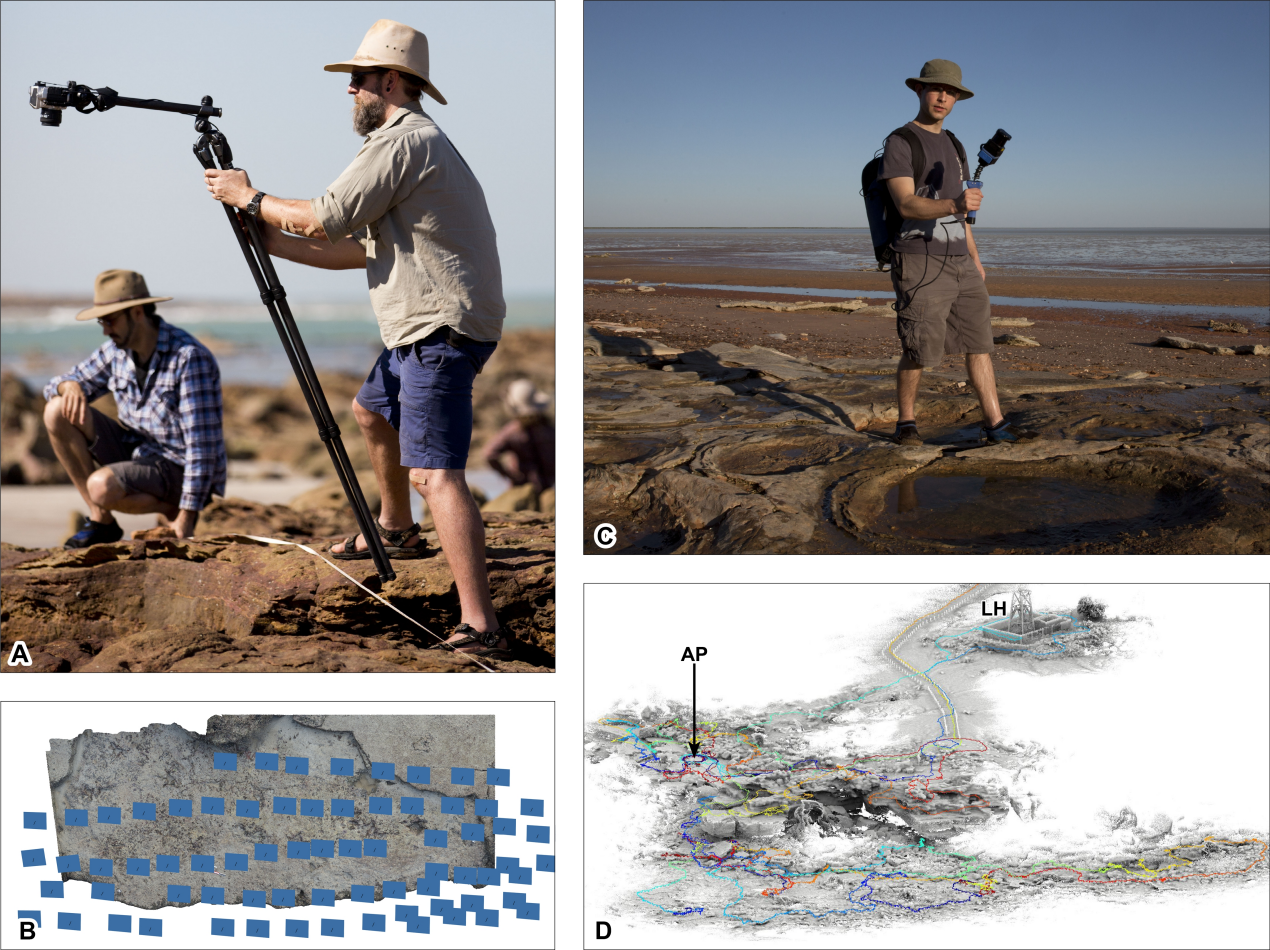
Ground-based data acquisition techniques used on Broome Sandstone dinosaurian tracksites. (A) Ground-based photography (left to right: AR and SWS). (B) photogrammtery-derived orthophotographic mosaic of the track-bearing platform UQL-DP56-4, -5 and -9 (blue rectangles indicate spatial position of the camera). (C) ‘Zebedee’ handheld ground-based laser scanning (RZ). (D) point cloud of Minyirr (UQL-DP56). Colored line indicates path taken whilst scanning. Area of B and C equals approximately 20 m^2^ and 4 ha, respectively. Abbreviations: AP, Anastasia’s pool; LH, Lighthouse. Photograph credit (A, C): Damian Kelly.

**Figure 5:**
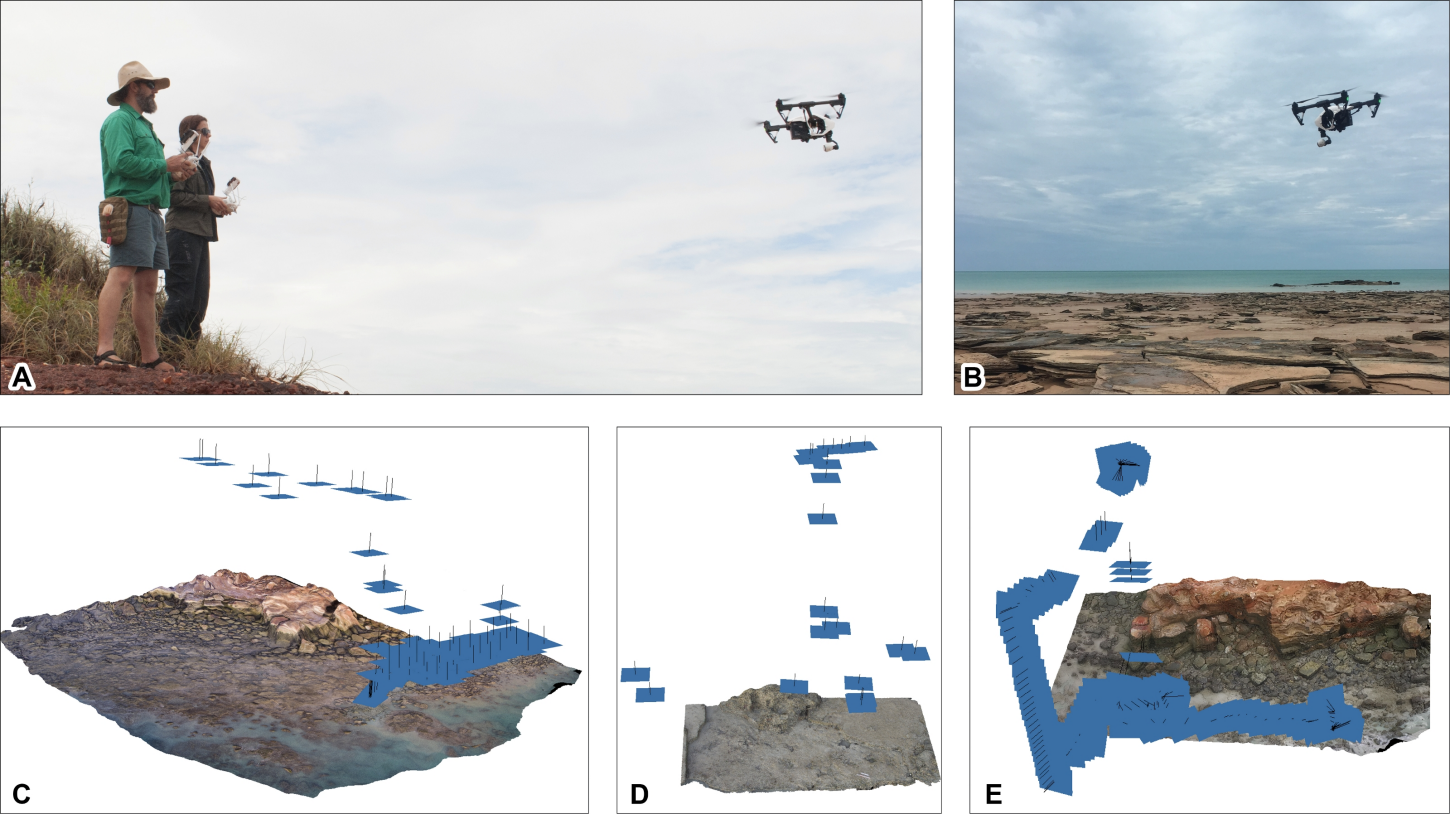
UAV aerial-based photographic data acquisition used on Broome Sandstone dinosaurian tracksites. (A) Pilot (L Pollard, UQ) and camera controller (SWS) with the UAV. (B) UAV over Broome Sandstone tracksite. (C) point cloud of Minyirr (UQL-DP56) derived from photographs. (D) close-up of C showing the main platform with tracks (UQL-DP56-4, -5 and -9). (E) point cloud of Minyirr (UQL-DP56) derived from video images. The blue rectangles indicate spatial position of the camera. Area of C, D and E equals approximately 1.8 ha, 20 m^2^ and 1.1 ha, respectively.

**Figure 6:**
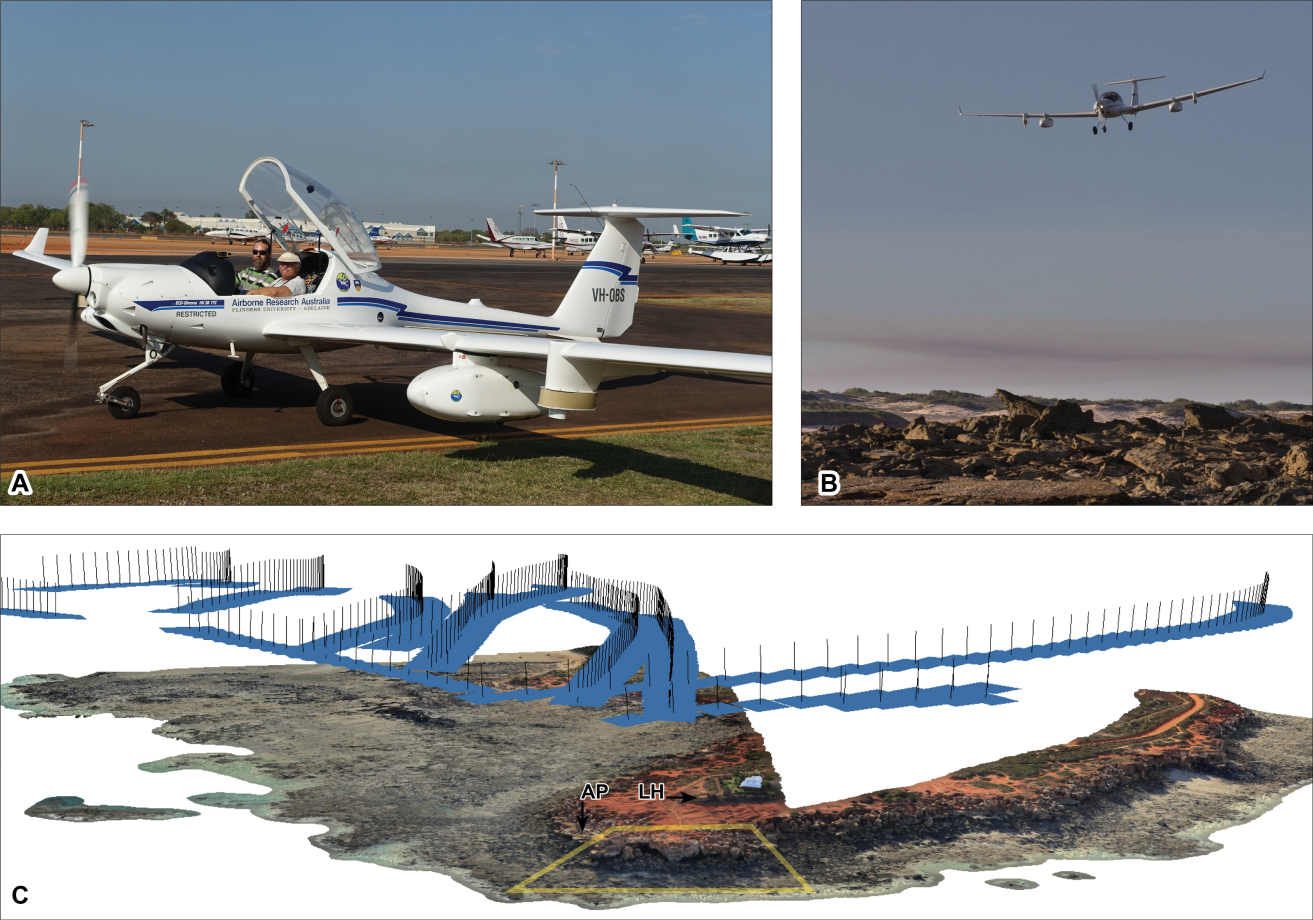
Photographic and laser data acquisition methods used from manned aircraft on Broome Sandstone dinosaurian tracksites. (A) Diamond Aircraft ECO-Dimona with SWS and JH (pilot). (B) ECO-Dimona over tracksite undertaking laser scanning and photography. (C) point cloud of the intertidal zone at Minyirr, derived from photographs. The blue rectangles indicate spatial position of the camera and the path taken by the aircraft. The yellow outline indicates the portion of Minyirr (UQL-DP56) that was the focus of the current study (2.4 ha). Note that laser scanning occurred simultaneously and continuously during photographic data acquisition. Total area of C equals approximately 13 ha. Photograph credit (B): Damian Kelly.

Dinosaurian tracks at Minyirr occur within the lower-most (seaward) exposures of Broome Sandstone, on *in situ* and *ex situ* intertidal rock platforms surrounding the main promontory. The intertidal zone at Minyirr covers an area of approximately 32 ha (∼320,000 m^2^; Fig. 2B). There are four main areas where tracks are concentrated within the general Minyirr area, each of which have been assigned University of Queensland Dampier Peninsula field locality numbers (UQL-DP56, 59, 61 and 63). This study focuses on the smallest tracksite, UQL-DP56 (hereafter referred to as DP56), which covers an area of approximately 2.4 ha (i.e., 24,000 m^2^; see Fig. 2B). The tidal range at Minyirr can exceed 10 m, such that direct examination of the dinosaurian tracks can only occur at extremely low tides. Data used in this study were collected during spring tide events in 2014 (September) and 2015 (March and June). Since commencing our study of the Broome Sandstone dinosaurian ichnofauna in 2011, we have identified six discontinuous track-bearing surfaces at DP56. Of these, four have not been completely lost to erosion (Fig. 3), with three containing theropod tracks formed (Figs. 3A–3F) and one set formed by a sauropod trackmaker (Figs. 3G and 3H). Within this study the different ground-based (Fig. 4) and aerial-based (Figs. 5 and 6) techniques were compared in their respective level-of-detail at the small-scale and large-scale. To highlight the outcomes of the data acquisition techniques for small-scale level-of-detail we have focused on the DP56 track-bearing platform that preserves many theropod tracks (DP56-4, −5 and −9; Figs. 3E–3F) that together comprise at least thirteen tracks. The acquisition large-scale level-of-detail data focused on encapsulating all of the track-bearing platforms (tide permitting) and the surrounding landscape.

### Photogrammetry data acquisition and processing

Four different photogrammetry methods were employed: one ground-based (Figs. 4A and 4B) and three aerial-based (Figs. 5 and 6). The latter involved acquiring photographs and videography from an Unmanned Aerial Vehicle (UAV; Fig. 5) and a manned aircraft (Fig. 6). In all of these approaches the surface structures were digitally photographed in a series of overlapping images in typically nadir (straight down) perspective under natural light conditions in order to record the parallax of the tracksite surfaces. The GPS metadata used in during ground- and UAV aerial-based photograph acquisition was not sensitive enough to distinguish positions less than 4 m apart and was subsequently disabled on the respective devices. RAW photographs were converted to JPEG using Adobe Photoshop CS6 (version 13.0 x64), or to PNG-24 file format when acquiring images from video. Converted images were then added to Agisoft PhotoScan Professional Edition (version 1.26 build 2038 64 bit) to generate DSMs (.ply, .obj, and .laz files) and orthophotographic mosaics (.png files). DSM’s were colorized using an ambient occlusion illumination in CloudCompare (version 6.0 Mac OS 64 bit) providing a ‘shaded’ visualization. False-color elevation maps and contour lines were created using Paraview (version 3.98.1 Mac OS 64 bit).

#### Ground-based photogrammetry

The surfaces of the *in situ* and ex situ track-bearing platforms were cleaned by hand to remove debris prior to taking photographs. Nadir and overlapping photographs (spaced approximately 25 cm apart) of the tracks and trackways were taken using a tripod mounted 16.2 megapixel Nikon Df with an AF Nikkor 24 mm f/2.8D autofocus lens. ISO, shutter speed and aperture were set manually depending on lighting conditions (Fig. 4A). 39-point dynamic area autofocus was used in conjunction with matrix metering, with the lens typically positioned approximately 1.5 m above the track surface, with photos taken using a remote shutter release to reduce camera shake.

#### Aerial-based photogrammetry: unmanned aerial vehicle (UAV)

The DJI T600 INSPIRE 1 (from this point onwards referred to as the UAV) was flown at low altitude (approximately 10 m) under the remote control of two operators (one pilot and one camera operator; Fig. 5A). Photographs of the surface were taken remotely every 1–5 s with the on-board 12.76 megapixel X3 gimbal-mounted digital camera (model FC350), which has a 20 mm (35 mm format equivalent) f/2.8 lens. Photographs were taken under natural lighting conditions (Figs. 5C and D 5), using auto settings for ISO, shutter speed an aperture. 4K Video footage (4,096 × 2,160 at 25 fps) was taken when the UAV was flown at low altitude (typically 4–120 m; Fig. 5E). Selected segments of the video MOV file format were imported into Adobe Photoshop CS6 to allow for the extraction of every 12th frame, at which automatic image downsampling occurred.

Regions containing ‘non-stationary objects’ (e.g., people, waves, etc.) were erased prior to the images being saved as PNG files, and subsequent addition to Agisoft PhotoScan for DSM creation.

#### Aerial-based photogrammetry: manned aircraft

In this study, the aircraft operator (JMH, Airborne Research Australia) flew over DP56 using the Diamond Aircraft HK36TTC-ECO-Dimona aircraft (from this point onwards referred to as ECO-Dimona; Fig. 6). Nadir photographs of the ground surface were automatically taken every one second using a Canon EOS 5D MkII digital camera (21 Megapixel) with a fixed 34 mm lens mounted to the aircraft’s wing pylon unit. For some of the flights, a GoPro Hero3+ was also carried, and a Sony DSC RX100M3 was also used for oblique photographs from the aircraft’s cockpit. In the following, only data obtained from the Canon EOS DSLR will be discussed and presented. The images were optimized using the DxO OpticsPro 10 software, then adjusted and rotated using ImageMagick Version 6.4.9. EXIF data was extracted using the ExifTool utility.

The ECO-Dimona is in essence a motor glider and represents an ideal platform for this project due to its excellent capabilities of slow flight (i.e., 35 m/s = 70 kts = 130 km/h) at low altitude (between 25 m and 180 m above the surface). A special dispensation from the Civil Aviation Safety Authority was attained as the legal requirement to perform such low altitude flights. The design features of ECO-Dimona, such as its very low noise footprint and excellent visibility from the cockpit, ensured that flights were executed with minimum disturbance to the people and wildlife on the beaches and surrounding areas. This is particularly the case at Minyirr, which occurs within an environmentally and culturally sensitive area that receives high tourist visitation.

### Laser data acquisition and processing

In this study of DP56, two different laser-scanning methods were employed: one ground- and one aerial-based approach (Figs. 4C and 6, respectively). Both techniques use lasers that are eye safe and require neither the use of safety glasses or safety barriers on the ground.

#### Aerial-based laser: manned aircraft

During the identical manned flights outlined above for ‘aerial-based photogrammetry: manned aircraft’ (Fig. 6), the ECO-Dimona was equipped with a full waveform resolving small footprint Airborne Laser Scanner (ALS; Riegl Q560, lidar pulse rate of 240 kHz, set to 135 lines per second, wavelength = 1,550 nm). This was paired with a tactical grade IMU/GPS system (a Novatel SPAN/LITEF LCI) allowing survey-grade georeferencing of the lidar point clouds. The multiple flight-passes provided up to 50 ground returns per square metre that allowed very high-density lidar point clouds with a scan area of approximately 110 m wide.

The lidar and IMU/GPS data was initially processed using a combination of Riegl proprietary software and Airborne Research Australia in-house software packages Routines for Airborne Scanner Processing (RASP Version 0.94.1657), Routines for the Processing of Meteorological Research Flights (RAMF Version 12.1.136) and Portable Pre-Processor (PPREP Version 1.3.52). Further processing utilized the LAStools library (https://rapidlasso.com/lastools/) and the GlobalMapper V13 to V17 software package. 30 cm-DSMs (laz and kmz files) were produced from single and multiple overpasses.

#### Ground-based laser: ‘Zebedee’ 3D mobile mapping system

Ground-based laser scans of DP56 acquired using the Zebedee 3D mobile mapping system (Bosse & Zlot, 2013; Zlot & Bosse, 2014; Zlot et al., 2014). For its primary sensor, Zebedee uses a 2D Hokuyo UTM-30LX-F laser scanner, which measures distances to surfaces at a 100 Hz scan rate with 41,600 maximum samples per second (i.e., some measurements are not reflected back, including those rays that are transmitted towards the sky or absorbed by water). The laser scanner is contained in a rigid housing with a MicroStrain 3DM-GX3 MEMS inertial measurement unit (IMU) that provides measurements of accelerations and rotational rates. The sensor housing is spring-mounted to a handheld grip such that as the operator walks the terrain, the spring is designed to allow the laser scanner to rotate outside the nominal scanning plane, thereby resulting in a 3D field of view. A USB GPS receiver (product/model number BU353-S4), mounted on the operator’s backpack, was used concurrently to enable geo-referencing of the results. Field-scans were typically limited to periods of 10–15 min, with areas having single, or more typically, multiple overpasses. CSIRO in-house software packages were used to process the acquired data and in the creation of dense point clouds (.laz and .ply files).

### 2D Map Generation

Google Earth Pro (version 7.1.5.1557) served as a platform to combine photogrammetry and aerial-based laser scan data. Placement of the laser data acquired from the ECO-Dimona (.kmz files) was automatic when opened in Google Earth Pro. Orthophotographic mosaics derived from the ground-, UAV aerial-, and ECO-Dimona aerial-based photogrammetry methods were downsampled using Adobe Photoshop CS6 (version 13.0 x64) and then overlaid in Google Earth Pro. These were manually repositioned, oriented and scaled to conform to the terrain of the kmz files and served as a more detailed photographic guide (compared to satellite imagery) of the track-bearing horizons in the context of landscape.

## Results

**Figure 7:**
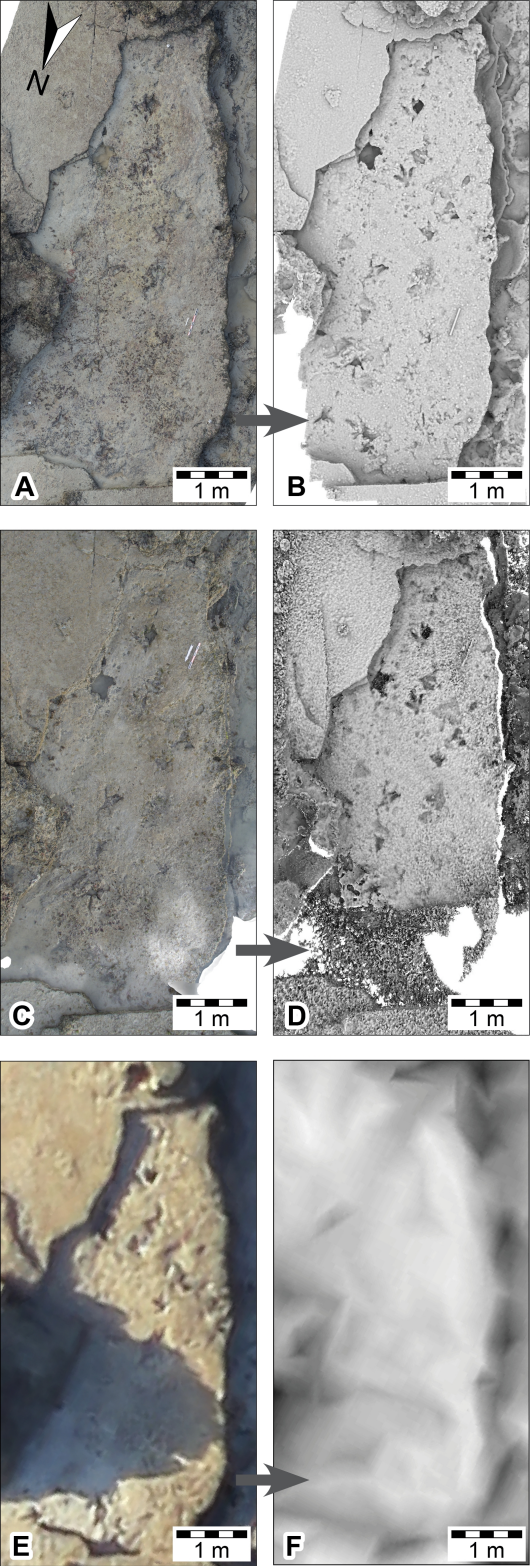
DSMs of *Megalosauropus broomensis* trackways (UQL-DP56-4 -5 and -9) at Minyirr derived from various photographic acquisition methods, over an area of approximately 20 m^2^. (A, B) ground-based models (orthophoto and ambient occlusion illumination, respectively). (C, D) UAV aerial-based models (orthophotographic mosaic and ambient occlusion illumination, respectively). (E, F) manned-aircraft, aerial-based models (orthophotographic mosaic and ambient occlusion illumination, respectively).

**Figure 8:**
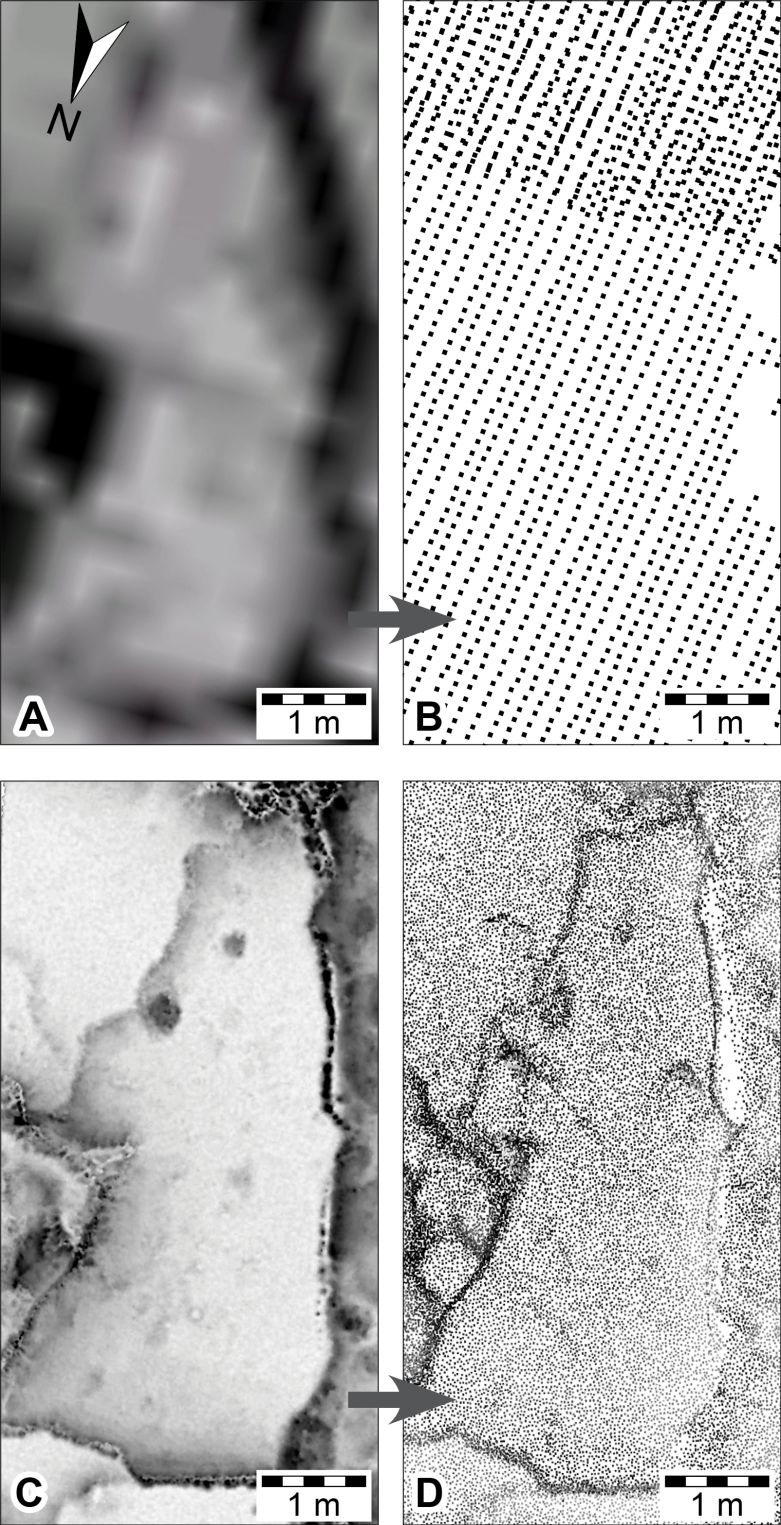
DSMs of *Megalosauropus broomensis* trackways (UQL-DP56-4, -5 and -9) at Minyirr derived from various laser acquisition methods, over an area of approximately 20 m^2^. (A, B) manned-aircraft aerial-based models (mesh and point cloud, respectively). (C, D) ground-based models (ambient occlusion illumination and point cloud, respectively).

**Figure 9:**
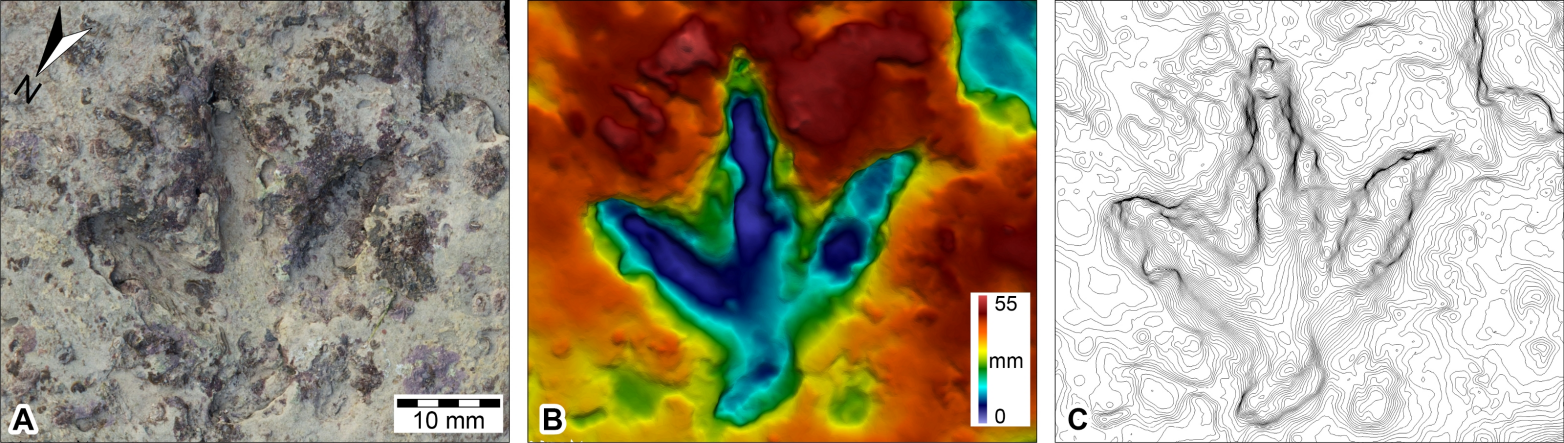
DSM of *Megalosauropus broomensis* UQL-DP56-4(lp3), Lower Cretaceous (Valanginian–Barremian) Broome Sandstone, Minyirr, Western Australia. DEMs derived from ground-based photogrammetry. (A) orthophotographic mosaic, (B) elevation map, (C) contour map (height intervals of 1 mm).

**Figure 10:**
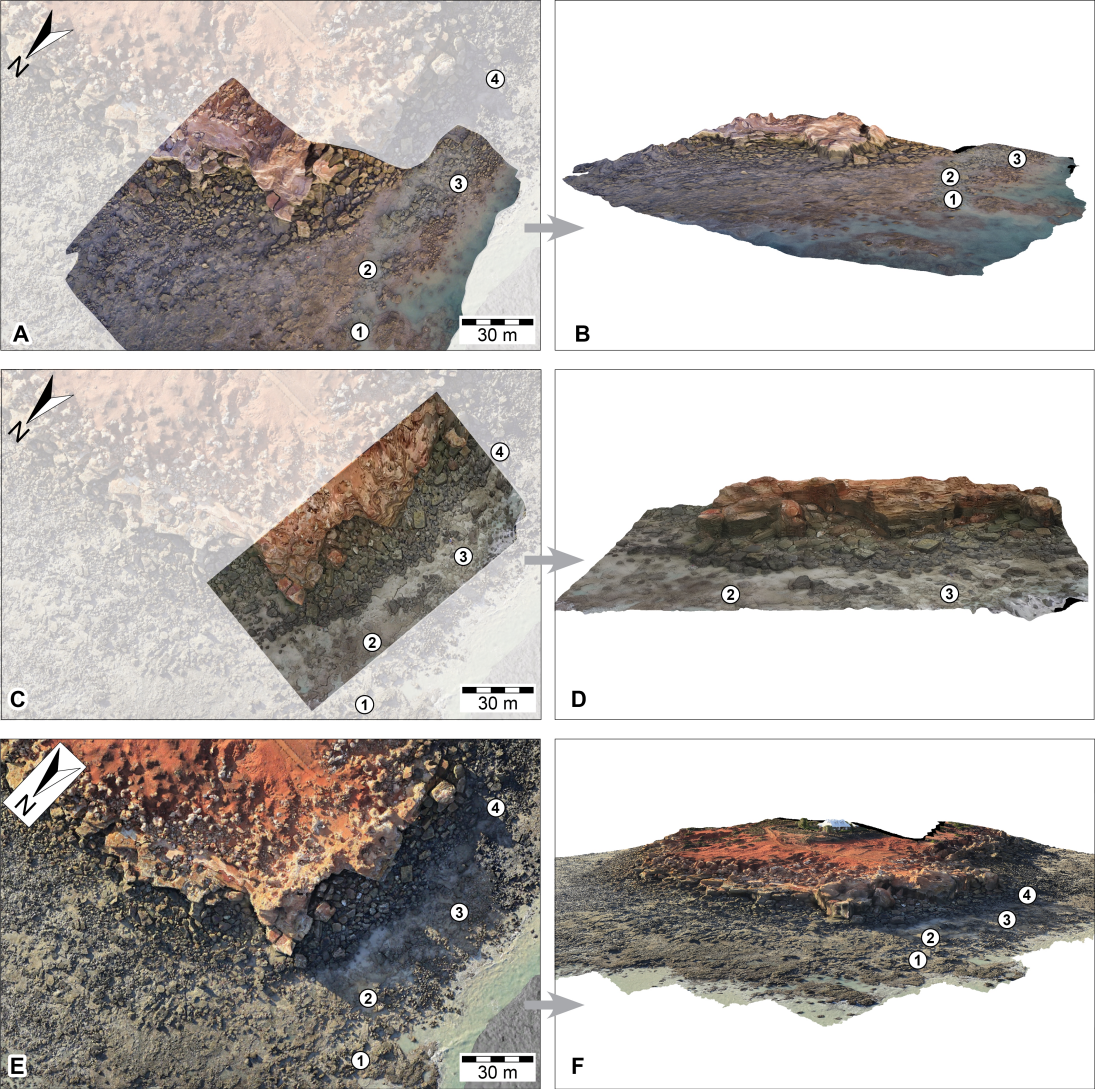
DSMs of the Minyirr dinosaurian tracksite (UQL-DP56) derived from various aerial-based photographic acquisition methods. (A, B) UAV aerial-based models (aerial and oblique view, respectively). (C, D) UAV aerial-based derived from video extracted images (aerial and oblique view, respectively). (E, F) manned-aircraft aerial-based models (orthophotographic mosaic in aerial and oblique view, respectively). All images are to the same scale with 30 m scale bar. Numbered place-markers denote the GPS position of track-bearing platforms: 1, UQL-DP56-4, 5, 9; 2, UQL-DP56-7, 8; 3, UQL-DP56-1, 2, 3; 4, UQL-DP56-6. A and C show areas that overlay an opaque image of C. The area of B, D, and F equals approximately 1.8, 1.1 and 13 ha, respectively.

**Figure 11:**
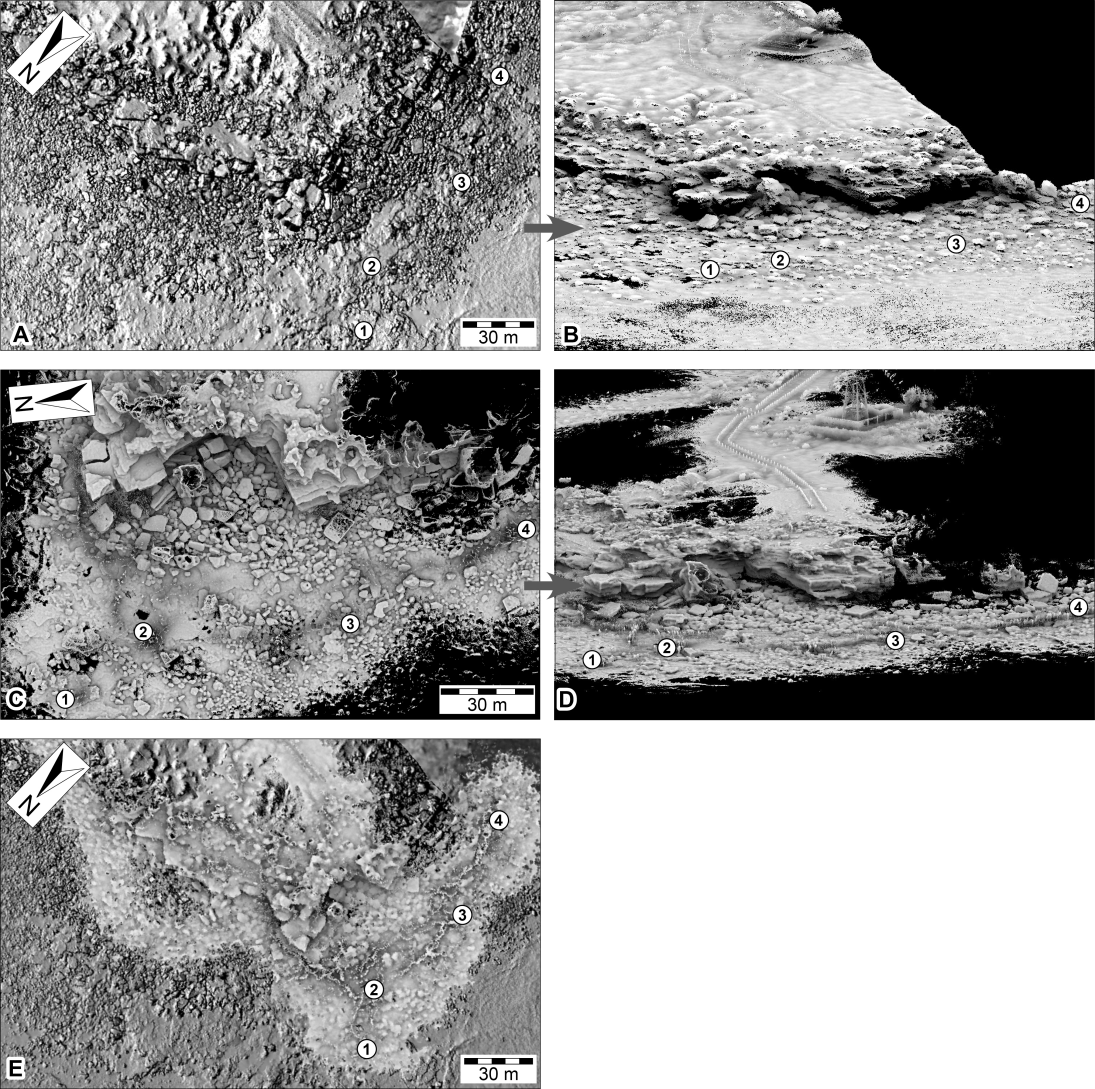
DSMs of the Minyirr dinosaurian tracksite (UQL-DP56) derived from various laser acquisition methods. (A, B) manned-aircraft aerial-based models (aerial and oblique view, respectively). (C, D) Zebedee handheld ground-based models (aerial and oblique view, respectively). (E) Data from C overlain on data from A. Numbered place-markers denote the GPS position of track-bearing platforms: 1, UQL-DP56-4, 5, 9; 2, UQL-DP56-7, 8; 3, UQL-DP56-1, 2, 3; 4, UQL-DP56-6. (A) and (C) images overlay an opaque image of (C).

High-resolution digital data of DP56 were acquired from each surface-based method (Table 1). Small-scale data for the platform that preserves two theropod trackways (DP56-4 and DP56-5; Figs. 7–9) was acquired using all the remote sensing techniques (Table 1), except for UAV video aerial-based photogrammetry during which time the platform was still submerged. Large-scale data for DP56 (Figs. 10 and 11) was acquired using all the remote sensing techniques, except for the ground-based photogrammetry. For the area covered, linear distance between points (also affected by the merger of multiple data scans), and the structural completeness (a function of line-of-sight perspective) varied between the techniques. Additionally, the photogrammetric data differed according to the natural lighting conditions at the different times of data acquisition, image resolution, and ground resolution (Table S1).

**Table 1:** Details of the remote sensing techniques used to survey UQL-DP56, Minyirr, south of Broome, Dampier Peninsula, Western Australia.

Method (Photogrammetry or LASER)	Coverage area (sq m)	Data collection duration	Processing time (iMac 3.5 GHz Intel Core i5 processor, AMD Radeon R9 M290X 2048 MB Graphics)	Dense point cloud number	Point density (pt/ sq m)	Average linear distance between points (=1/(square root of [pts/sq m]) in metres	Average linear distance between points (mm)
PG-ground-based	22.7	12 m	3 h 2 m	53,728,920	2,366,912.78	0.00065	0.65
PG-aerial UAV photo	44	<1 m	52 m	4,128,800	92,991	0.00328	3.28
PG-aerial UAV photo	15,200	8 m	3 h 1 m	41,061,249	2,701.40	0.01924	19.24
PG-aerial UAV video	8,780	8 m	2 h 21 m	2,343,893	266.96	0.06120	61.20
PG-aerial Plane	124,000	<2 m	1 h 5 m	4,301,633	34.69	0.16978	169.78
LASER-ground-based	42,062	2 h 49 m		102,182,615	2,429.33	0.02029	20.29
LASER-aerial	914,694	<8 m		52,241,139	57.11	0.13232	132.32

### Ground-based photogrammetry

Ground-based photogrammetry provided the greatest detail of all the remote sensing techniques utilized in the study, with an average of over two million points per square metre or 0.65 mm distance between data points (Table 1). Photographs (*n* = 77) were taken in five passes over the track-bearing platform (and the immediate surrounding area) with a coverage of ∼23 m^2^, in a time of ∼12 min. The lighting and tidal conditions were ideal for this mode of data collection. The subsequent DSM provided highly detailed structural data that enabled track and trackway parameters to be easily discernible (Figs. 7A–7B, and 9).

### UAV aerial-based photogrammetry

After a number of earlier flights were aborted because of rain, two successful UAV flights were made at DP56 for the data acquisition of photographs and video. Both of these subsequent flights took place under the suboptimal conditions of high pedestrian traffic, low light conditions (at sunset for photographic capture; and heavily overcast for video capture), and in the case for the video capture photogrammetry during occasional light precipitation and during tide times that meant some of the tracks were not subaerially exposed.

#### Photographic UAV aerial-based photogrammetry

For investigation of the small-scale level-of-detail, twenty photographs were used in the creation of the single theropod track platform (Figs. 7C and 7D). These images were derived when the UAV rose above the platform during the near-completion of the track surface cleaning (Fig. 5D), although there is some obstruction of the surface by the cleaners and subsequently required masking for post processing. The surface coverage was small (44 m^2^) yet retained high surface detailed (second only to the ground-based photogrammetric method) with an average of approximately 3 mm spacing between points (Table 1). With the exception of the ‘cleaner’-obscured surface region, track and trackway features were easily visible. The manual operation of the UAV obtained data rapidly (∼8 min; see Table 1) and covered an area of approximately 1.52 ha (15,200 m^2^) of the DP56 tracksite (Figs. 5C and 10A–10B). Photographic data was obtained for most, but not all currently (as of 2015) existing track-bearing platforms at this tracksite, due to failing light conditions. More importantly, the photography under the already fading light conditions resulted in most of the images being slightly out of focus. Despite these issues, the resultant DSM (derived from 166 photographs) provided reasonably good topographical details of the tracksite (average linear distance between points of 19 mm; Table 1). It is likely that better detail could be obtained under more optimal lighting conditions.

#### Video UAV aerial-based photogrammetry

The second UAV flight filmed an area of approximately 1.5 ha (15,200 m^2^) of DP56 for approximately eight minutes. The high pedestrian traffic during the data collection session necessitated their digitial removal leading to an increase in image manipulation time prior to creating the surface model. Despite these challenges, reasonably good topographical resolution was obtained for DP56 using video data collection (average of 61 mm linear distance between points; Table 1; 10C–10D). Large rocky outcrops and the cliff line are easily recognisable from the DSM that has perspectives from the sides as well as above. However, small structures such as tracks are indistinguishable.

### Manned aircraft aerial-based photogrammetry

The aerial-based photogrammetry via the manned aircraft took place during extremely low tide, under excellent lighting conditions, with very low pedestrian traffic (Figs. 7E–7F and 10E–10F). DP56 was flown over eight times using a flight path that also encompassed coastal exposures of Broome Sandstone extending west into Roebuck Bay. This flight took around 45 min with approximately 2,800 photographs taken during this time. Data collection at Minyirr was less than two minutes, during which time 93 photographs were taken that covered 12.4 ha (124,000 m^2^). Although the 3D model derived from these photographs is relatively sparse (∼170 mm distance between points) an excellent perspective is obtained at the landscape scale of the intertidal platforms adjoining tracksites, cliff-line, and surrounding geography. For tracks measuring <50 cm in length, information is not resolved.

### Aerial-based laser scanning

ECO-Dimona aerial-based laser scanning occurred concurrently with the aircraft’s photographic data collection. The nadir position for scans and high elevation provide an advantageous broad perspective of the DP56 tracksite terrain with an average distance between points being 132.32 mm (Table 1). Ground registered scan points obtained during the flight provide little detail of the 3D surface structure when viewed closely such that the tracks from DP56-4-5 are not discernable (Figs. 8A–8B), although it should be added that tracks greter than 50 cm in length are recognised. Surfaces not in the nadir field-of-view (e.g., below overhangs) were not registered in these scans.

### Ground-based laser scanning

The ground-based laser scanning via the Zebedee mobile mapping system was performed for multiple tracksites along the Dampier Peninsula over a period of six days under ideal weather conditions (Figs. 9C–9D and 11C–11D). Maximum low tide exposures of the intertidal zone at DP56 were scanned during a single session, and the upper exposures of Broome Sandstone and surrounding landscape scanned at high tide, resulting in an overall coverage of approximately 4.2 ha (42,060 m^2^). The resolution was very good for the terrain with good scan resolution (an average of 20.29 mm distance between points) that is nearly the same as the sub-optimal conditions for the UAV large-scale photogrammetry (Table 1); however, only surfaces within a distance of about 15 m of the scanner are typically usable. The track-bearing platforms are recognizable, but the position of the shallowly impressed tracks could not be resolved (Figs. 9C–9D).

### Mapping

**Figure 12:**
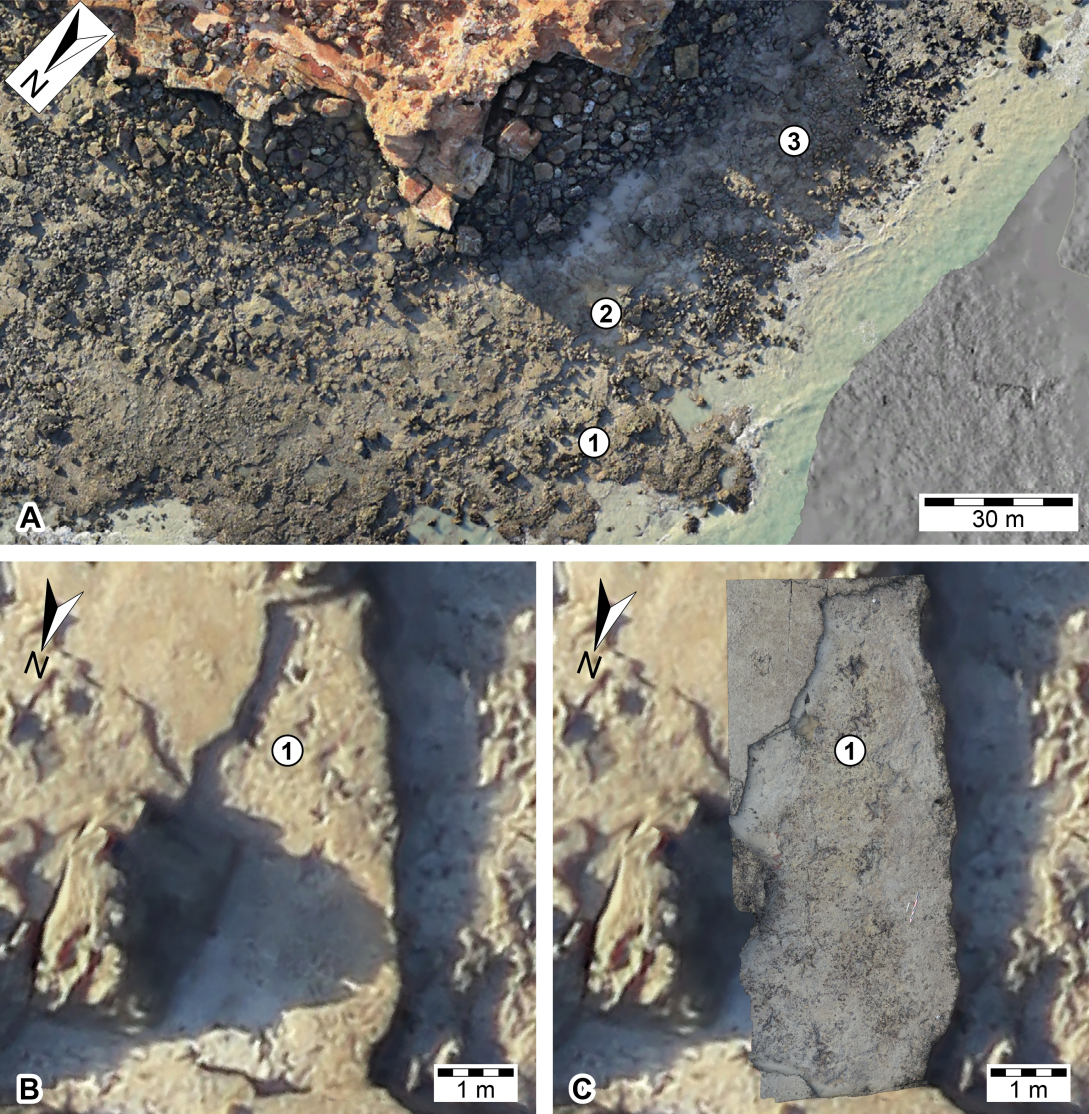
Overlay of different ‘level of detail’ datasets of the Minyir dinosaurian tracksite (UQL-DP56). (A) orthophotographic mosaic of the landscape derived from a manned-aircraft. (B) close-up of the main platform with theropod tracks (UQL-DP56-4, -5 and -9) derived from a manned-aircraft. (C) overlain orthophotographic mosaic of the main platform with theropod tracks (UQL-DP56-4, -5 and -9) derived from ground-based photogrammetry. Numbered place-markers denote the GPS position of track-bearing platforms: 1, UQL-DP56-4, 5, 9; 2, UQL-DP56-7, 8; 3, UQL-DP56-1, 2, 3.

2D maps were successfully created within Google Earth Pro by importing ECO-Dimona laser-scan derived .kml files (Fig. 11A) and overlaying the Zebedee laser-scan .png file (Fig. 11E) and the photogrammetry derived orthophotographic mosaics (Fig. 12). The automatic placement of each file type as separate layers within Google Earth Pro enabled data fields to be view individually or in combination. The elevation views that transitioned between the ground sourced track/trackway data sets and the aerially sourced data sets were not seamless. Specifically surface details were lost at: ∼30 m altitude for UAV aerial-based photogrammetry; ∼40 m altitude for ‘video’ UAV aerial-based photogrammetry; ∼70 m altitude for both ECO-Dimona aerial-based photogrammetry and laser scanning.

**Figure 13:**
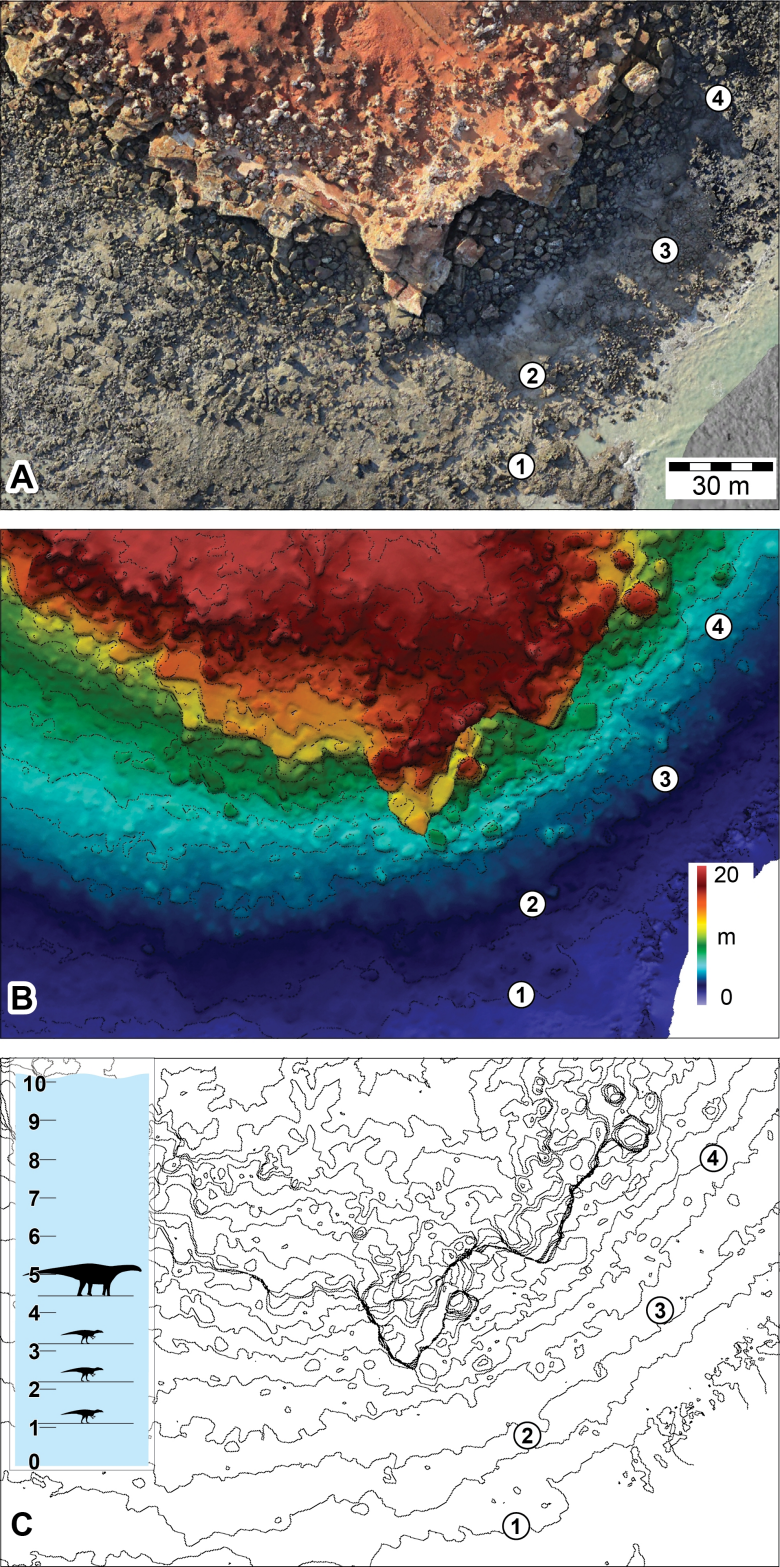
Use of DSMs to ascertain vertical positions of the track-bearing horizons at the Minyirr dinosaurian tracksite (UQL-DP56). (A) Aerial view of tracksite as orthophotographic mosaic. (B) Elevation map of the tracksite. (C) Contour map of the tracksite (isoline intervals every 1m vertically), with an insert showing relative vertical position of main track-bearing horizons. Units are in meters. Silhouettes represent hypothetical trackmakers. Numbered place-markers denote the GPS position of track-bearing platforms: 1, UQL-DP56-4, 5, 9; 2, UQL-DP56-7, 8; 3, UQL-DP56-1, 2, 3; 4, UQL-DP56-6.

The DSM created from the ECO-Dimona aerial-based photogrammetry also allowed for elevation and contour filters to be applied in order to aid visualization of the tracksite topography (Fig. 13). The relative vertical position of each of the *in situ* track-bearing surfaces is apparent (Fig. 13C), but how these relate to their relative stratigraphic position awaits further investigation (the dip at Minyirr in negligible (1–2 degrees), so the topographic height closely approximates stratigraphic level).

## Discussion

Research on the dinosaurian tracksites of the Broome Sandstone involves overcoming a number of non-trivial challenges, including, but not limited to the bi-daily submergence of tracks by significant depth of water, and the broad distribution of the tracksites over 100+ km of often remote and hard to access coastline. The photogrammetric and laser scanning techniques used here in the study of DP56 demonstrate that these challenges can be successfully addressed and, in the process, yield high-fidelity 3D digital data from the level of sub-track details (Fig. 9) to large scale landscapes (Figs. 10–11).

Data acquisition for DP56 using the various types of remote sensing techniques outlined in this study was undertaken relatively quickly (seconds to hours) and with a broad coverage (tens–100’s of thousands of square metres). Considering the broad area (approximately 100+ kilometres) over which dinosaurian tracksites in the Broome Sandstone occur, and the brief window during which many of these sites are accessible, remote sensing techniques mean that field resources can be effectively utilized, with much of the time-consuming processing and analysis of data taking place offsite.

Image based photogrammetry is extremely flexible and the coverage, data density and precision of the final products are complex functions of the sensor pixel count, sensor pixel size, sensor focal length and the geometry of data acquisition as well as aspects of the image processing. Detailed analyses of the relative performance of any photogrammetric network are possible and should be performed before field mapping is commenced to ensure that the data acquisition is optimised for the purpose.

As expected, of the photometric methods used in this study, the ground-based photogrammetry provides highest surface structure resolution. The detailed DSM generated through this method are ideally suited to computational analyses of the track and trackway characteristics, with fully textured models providing an accurate visualization of the surface appearance and topography present at the time of data collection. This approach is the most relevant in the direct study of dinosaurian ichnites, for digital archiving the tracks (of particular relevance for their National Heritage Listing) and to monitor track surface preservation (under near constant erosional forces within the Dampier Peninsula intertidal zone).

DSM’s derived from ground-based photogrammetry have become increasingly popular within the field of dinosaurian ichnology. This is very likely because of the simplicity associated with the ease of point-and-shoot camera operations as the basic means of data collection. As a consequence this approach has been used for track data collection (Thulborn, 1997; Matthews & Breithaupt, 2001; Breithaupt, Matthews & Noble, 2004; Matthews, Noble & Breithaupt, 2006) and documentation (Bates et al., 2008; Petti et al., 2008; Romilio & Salisbury, 2014; Pond et al., 2014; Salisbury et al., 2014), ichnotaxonomic assessments (Pond et al., 2014; Lallensack, Van Heteren & Wings, 2016; Salisbury et al., in press), and for demonstrating track sequence formation (Xing et al., 2015).

Photographic data processed using UAV aerial-based photogrammetry also provides an accurate digital record the surface structures in high fidelity. Under the sub-optimal conditions, the associated UAV-derived point clouds were five to ∼30 times sparser (i.e., fewer points) than those from ground-based photogrammetry (average distance between points being 3.28–19.24 mm versus 0.65 mm, respectively). The density of the point cloud being related to proximity to target: at a greater ranges, a pixel covers a larger angular area of the field of view (given all other things are equal). It may also relate to the number of overlapping images, particularly when considering the acquisition of relatively few useable images acquired on this occasion with the UAV when compared with the ground-based method (*n* = 20 and 77, respectively). Yet in practical terms, 3D model densities of very high values (such as those obtained here for ground-based methods) are generally not a requisite in dinosaurian ichnological studies.

The process of preparing the track-bearing surfaces (i.e., the removal of marine debris and water) prior to them being photographed, using either ground- or UAV aerial-based methods, is a necessary part of good data acquisition for most dinosaurian tracksites on the Dampier Peninsula. Although it can be a time-consuming process, the results are improved in instances when *in situ* surface cleaning is undertaken, particularly so for ground-based photogrammetry. For tracksites in the intertidal zone, the presence of water, sand and other types of intertidal debris can greatly obscure the preserved details of the exposed sedimentary surfaces and any associated ichnites. The time required for surface preparation is typically not a problem for relatively small track-bearing platforms (e.g., those with a total area of <50 m^2^), such as those at DP56. Cleaning larger tracksites is much more difficult or even impossible for some of the more remote locations surveyed by the manned aircraft.

Other Broome Sandstone tracksites with large track-bearing platforms could also benefit from having the surface structure recorded in detail. The time-cost of data collection can be an important consideration particularly since the subaerial exposure of the sites is limited by tides. The UAV aerial-based photogrammetry provides a method where detailed data can be acquired with a broad coverage (m^2^–ha+ range) relatively rapidly. In the case of DP56, photographic data was collected across 1.5 ha (15,200 m^2^; Table 1) within eight minutes. The resultant DSM provided a highly accurate surface model of much of this tracksite, and performed remarkably well when considering a number of conditions that limited the quality of the data collected (poorly focused images due to low light when tracks were subaerially exposed) and the amount of data that was collected (single pass over the site when it became too dark). With increasing payloads UAVs can carry better cameras and have the potential make in-field handheld ground based image acquisition unnecessary.

Given the limited number of flat and dry surfaces, the DJI INSPIRE 1 UAV had operational advantages over traditional ichnological mapping techniques. As a quadcopter, it is able to perform vertical takeoff and landings within a relatively small area (i.e., <1 m^2^)—a capability that proved invaluable at Minyirr with its limited number of flat and dry surfaces. The gimbal configuration of the INSPIRE 1 stabilizes the camera platform while images are captured, even with moderate (<30 km/h) wind gusts. During flight, operators can be distant (tens to hundreds of meters) from the surfaces being photographed. This makes it possible to conduct broad sweeps of tracksites from a safe location, even if the site is located close to water or on a partially submerged offshore reef.

A DSM of much of DP56 was successfully obtained from UAV video-derived data (Figs. 10C–10D). One of the main advantages of using this technique to acquire photos for DSMs is the speed at which a large dataset can be acquired (e.g., ∼10,000 potential images from eight minutes of footage at 24 fps). However, the photogrammetry software we used does not use video data directly, instead this information needs to be first converted into multiple individual images, and then a selection of images is required for DSM creation. Limitations associated with our processing the image through Photoshop was the automatic downsampling of images during the video-to-image conversion process, although other software may be available that does not reduced image resolution. More considerable is the time-consuming task of selecting images from the large video data set, and the removal of areas with moving objects (e.g., people, waves, etc.) prior to model creation. As it stands, the use of video is not our preferred approach for when data is to be converted to 3D models. Nevertheless, for tracksites where time is very limited, UAV video is still a viable option, particularly under good lighting conditions.

Of the remote sensing techniques employed, the ECO-Dimona aerial-based concurrent photogrammetric and laser scan data acquisition provided the greatest coverage in terms of overall surface area. The data collected using this method is not restricted to the 2.4 ha (i.e., 24,000 m^2^) area of intertidal DP56 at Minyirr, but is instead part of a larger dataset of an area that includes, but that is not limited to, adjoining tracksites and landscape (e.g., 91 ha; Fig. 2B). This represents only a small section of data collected during a single flight lasting 45-minutes revisiting coastal regions from Roebuck Bay to Minyirr. During that particular flight, aerial-based photogrammetric and laser scan data for a total of 15 tracksites was collected. Subsequent flights along the Dampier Peninsula coastline acquired data for a total of 63 of the tracksites that we have recorded, discontinuously distributed across 100+ km.

At lower resolutions the data collected from the ECO-Dimona often does not detect tracks but the platforms that tracks are preserved on are recognizable (NB. Broome Sandstone tracks greater than 50 cm length are visible; see Hacker, 2015). As with the UAV acquired data, each track-bearing platform can be placed in the context of other platforms with their tracksite. Unique to the information collected from the ECO-Dimona is that the spatial perspective between multiple neighbouring tracksites and the surrounding geography is easily visualized. Additionally, freely available software such as Google Earth Pro provides a user-friendly framework of integrating the spatially extensive .kmz file data collected by the ECO-Dimona onto satellite images of Earth (Fig. 11A). This also permits the incorporation high-resolution, track and trackway data derived from photogrammetric approaches (i.e., as orthophotographic mosaics) tracksite information to become visualized at varying levels-of-detail within the same file (Fig. 9).

The DSMs created from data derived from the Zebedee handheld laser scanner provided excellent geometry of the terrain at DP56. Zebedee-derived DSMs were good for resolving surface structures at middle-scale detail within 15 m of the scanner position. The ability to walk around the terrain provides full control of the area covered (provided it is accessible on-foot), an ability to rescan areas in order to obtain greater detail of surface structures, and the option to scan from multiple viewpoints to improve the structural completeness of the surface. The qualities and scan resolutions (average linear distance between points of 20.29 mm) complement those of the ECO-Dimona laser data that can be merged for 2D map visualizations within Google Earth Pro or even as 3D maps within other software platforms (e.g., CloudCompare).

Prior to our team’s survey work, the available map data on the Minyirr dinosaurian tracks comprised stylized hand-drawn 2D outlines. The high accuracy and objective topographic recording of tracksite data across a range of spatial scales of our work provides a major advancement over other approaches traditionally used in ichnological studies. The use of remote sensing technologies in particular provides an important means of digitally conserving these important heritage sites, with the potential to monitor their changing preservational state over time.

The rapid data acquisition using the methods outlined herein is of tremendous benefit in the documentation of intermittently exposed tracksites. The information gathered from tracksite elevation maps can also be used to calculate the tidal level at which track-bearing horizons are submerged and exposed (Fig. 13C). Our fieldwork on the Dampier Peninsula usually takes place during tidal episodes that maximize subaerial exposures of the Broome Sandstone. Tracksite elevation maps provide an additional tool by which fieldwork efforts can be co-ordinated relative to fluctuating tidal conditions and in the future may provide an invaluable aid for public visitation of certain tracksites. Additionally, a major benefit of adopting field methods that rapidly acquire quantitative data is the freedom to conduct the bulk of analytical work remotely.

## Conclusion

The National Heritage Listed dinosaurian tracks of the Lower Cretaceous (Valanginian–Barremian) Broome Sandstone represent a highly diverse ichnofauna. Traditional scientific methods for documenting dinosaurian tracksites in this area have been severely limited due to non-trivial site access challenges. These include the remoteness of track site locations, the brief period during which track-bearing platforms are subaerially exposed, and the spatial extensiveness of the tracksites. Ground- and aerial-based photogrammetric and laser scanning remote sensing methods provide a novel approach for recording ichnological data associated with the Broome Sandstone. In this study, high fidelity digital data of the surface structures at DP56 was obtained across a wide range of scales of magnitude. The level-of-detail ranges from the sub-millimetre track morphology to visualizing and georeferencing the spatial relationship between tracksites. Additionally, the acquisition of large datasets from these remote techniques occurs very rapidly, providing a thorough record of the briefly exposed surface structures, and allows further analysis and interpretive research to be conducted off-site. The remote sensing approaches outlined herein provide significant advantages over traditional techniques and provide new opportunities for scientific exploration and digital conservation of important heritage structures. Additionally, such information has the potential for the temporal monitoring of the dinosaurian tracks. As plans for the ongoing management of the Dampier Peninsula’s dinosaurian tracks progress, 3D visualization will likely become an important means through which the broader public can experience these spectacular National Heritage listed landscapes.

##  Supplemental Information

10.7717/peerj.3013/supp-1 Table S1 Photographic details used to survey UQL-DP56, Minyirr, south of Broome, Dampier Peninsula, Western Australia

## Additional Information and Declarations

### Data Availability

The following information was supplied regarding data availability: The raw data is included in the manuscript in Table 1, with additional information presented in the Table S1.
