# Supplementary material for: A multidisciplinary approach to digital mapping of dinosaurian tracksites in the Lower Cretaceous (Valanginian–Barremian) Broome Sandstone of the Dampier Peninsula, Western Australia
Source: PeerJ. 2017 Mar 21;5:e3013. doi: 10.7717/peerj.3013 (PMC5363262; doi:10.7717/peerj.3013)
Supplement: Table S1 [file peerj-05-3013-s001.docx]

| Photogrammetry method | Image resolution (av) | Image resolution | Number of images | Ground resolution | Pixel size | Average camera height (m) |
| --- | --- | --- | --- | --- | --- | --- |
| Ground-based | 11 MB | 4928 x 3280 | 77 | 0.436 mm/px | 7.31 x 7.31 um | 1.5 |
| Aerial UAV image | 10 MB | 4928 x 3280 | 20 | 2.33 mm/px | 1.57 x 1.57 um | 6.3 |
| Aerial UAV image | 10 MB | 4928 x 3280 | 166 | 5.49 mm/px | 1.57 x 1.57 um | 15.1 |
| Aerial UAV video | 3MB | 1920 x 1080 | 238 | 6.1 cm/px | ? | 71.3 |
| Aerial Plane | 12 MB | 5616 x 3744 | 93 | 9.91 cm/px | 6.55 x 6.55 um | 150.0 |
